# Late normal tissue effects in the arm and shoulder following lymphatic radiotherapy: Results from the UK START (Standardisation of Breast Radiotherapy) trials

**DOI:** 10.1016/j.radonc.2017.10.033

**Published:** 2018-01

**Authors:** Joanne S Haviland, Mariella Mannino, Clare Griffin, Nuria Porta, Mark Sydenham, Judith M. Bliss, John R. Yarnold, the START Trialists' Group

**Affiliations:** a Institute of Cancer Research Clinical Trials and Statistics Unit (ICR-CTSU), Division of Clinical Studies, The Institute of Cancer Research, London, UK; b Società per l’Assistenza al Malato Oncologico Terminale (SAMOT), Palermo, Italy; c Division of Radiotherapy and Imaging, The Institute of Cancer Research, London, UK

**Keywords:** Breast cancer, Radiotherapy, Hypofractionation, Normal tissue effects, Lymph nodes

## Abstract

**Background and purpose:**

Adjuvant lymphatic radiotherapy (LNRT) is recommended for selected axillary node positive women with early breast cancer. We investigated whether hypofractionated LNRT is safe combined with similarly-hypofractionated breast/chest wall radiotherapy (RT).

**Material and methods:**

The Standardisation of Breast Radiotherapy (START) pilot, A and B trials randomised women with early breast cancer to schedules of 2.67–3.3 Gy versus 2.0 Gy fractions (control). RT adverse effects were assessed by patients using the EORTC QLQ-BR23 and protocol-specific questions, and by physicians. Rates of arm/shoulder effects were compared between schedules for patients given LNRT.

**Results:**

864/5861 (14.7%) patients received LNRT (385 START-pilot, 318 START-A, 161 START-B). Prevalences of moderate/marked arm/shoulder effects were low up to 10 years. There were no significant differences between the hypofractionated and control groups for patient- and physician-assessed symptoms in START-A or START-B. In START-pilot, adverse effect rates were higher after 13 fractions of 3.3 Gy, consistent with effects reported in the breast/chest wall (significant for shoulder stiffness, HR 3.07, 95%CI 1.62–5.83, *p* = 0.001).

**Conclusions:**

The START trial results suggest that appropriately-dosed hypofractionated LNRT is safe in the long-term, according to patient and physician-assessed arm and shoulder symptoms. These findings are consistent with those reported after the same schedules delivered to the breast/chest wall.

Four randomised trials enroling more than 7000 women with early breast cancer in the UK and Canada between 1986 and 2002 have demonstrated that hypofractionated radiotherapy (RT) schedules are as effective and safe as standard fractionation [1], [2], [3], [4], [5]. The three START trials [2], [3], [4], [5] tested fraction sizes >2 Gy delivered in 3 or 5 weeks against the international standard 50 Gy in 25 fractions over 5 weeks and showed non-inferiority for the hypofractionated schedules in terms of local tumour control as well as less toxicity relating to late normal tissue effects in the breast. Similarly, the Canadian trial [1] showed that 42.5 Gy in 16 fractions delivered over 3 weeks was non-inferior to the international standard schedule, although all women were axillary node negative and no lymphatic RT was given.

The results of these trials have been interpreted with various levels of caution in different countries. The 2016 consensus statements prepared by the UK Royal College of Radiologists states “There is no indication to use more than 15 fractions for the breast, chest wall or nodal areas for standard adjuvant treatment” [6]. A more restrictive adoption of hypofractionation is indicated in the 2011 American Society for Radiation Oncology (ASTRO) guidelines [7], which supported hypofractionated RT in selected patients but judged that there were insufficient numbers treated with irradiation of nodal areas in the randomised trials to form the basis for an evidence-based recommendation of hypofractionated lymphatic RT. Similar indications are given in guidelines from the German Society of Radiation Oncology (DEGRO) [8], the Italian Society of Radiation Oncology (AIRO) [9] and Cancer Australia [10].

Using a retrospective evaluation of prospectively collected data from three randomised trials, this paper aims to investigate patient- and physician-assessed late normal tissue effects in the arm and shoulder in women treated with lymphatic irradiation within the START trials testing hypofractionation in early stage breast cancer [2], [3], [4], [5], to determine if, as was found in the studies overall, appropriately-dosed hypofractionated RT is safe when applied to lymphatic areas.

## Materials and methods

The START-pilot trial (*n* = 1410, 1986–1998) was a phase III randomised trial in early breast cancer (T1–2, N0–1, M0) testing whether fewer larger fractions of post-surgical radiotherapy would be as safe and effective as the international standard schedule 50 Gy in 25 fractions of 2.0 Gy, and tested two hypofractionated schedules (42.9 Gy in 13 fractions of 3.3 Gy and 39 Gy in 13 fractions of 3.0 Gy, all given over 5 weeks) against this control [2]. Based on this trial, the START trials (1999–2002) were initiated consisting of two parallel trials: START-A (*n* = 2236) and START-B (*n* = 2215) to extend the testing of radiotherapy schedules using fraction sizes larger than 2.0 Gy in terms of locoregional tumour control, normal tissue effects, quality of life and health economic consequences in early breast cancer (T1–3, N0–1, M0) [3], [4], [5]. Patients were ineligible for trial entry if they required axillary radiotherapy after greater than a Level 1 axillary dissection or after >10 lymph nodes had been removed. START-A randomised between 50 Gy in 25 fractions of 2.0 Gy (control), 41.6 Gy in 13 fractions of 3.2 Gy and 39 Gy in 13 fractions of 3.0 Gy, all given over 5 weeks. START-B randomised between the same control schedule and 40 Gy in 15 fractions of 2.67 Gy over 3 weeks. A subset of centres in START-A and START-B participated in a quality of life patient-reported outcome measures (PROMS) study, which recruited 2208 women who had received breast conserving surgery [11].

In all three START trials lymphatic radiotherapy was permitted to the axillary chain and/or the supraclavicular nodes; the decision to give lymphatic radiotherapy was made before randomisation. Where lymphatic radiotherapy was recommended as part of standard of care, most commonly a minimum of 4 positive axillary nodes following axillary sampling or dissection, the planning target volume was supraclavicular nodes or axillary chain with a 1 cm margin. In two START-A patients prescribed radiotherapy to the breast and supraclavicular fossa and randomised to the 41.6 Gy schedule, the total dose administered to the supraclavicular fossa was reduced to 39 Gy because of a perceived concern about the sensitivity of brachial plexus to fraction size. Radiotherapy quality assurance was an integral part of the trials. Full details of procedures are described elsewhere [3], [4].

Late normal tissue effects in the arm and shoulder were assessed by physicians in all three trials and also by patients in START-A and START-B. Annual physician assessments of late normal tissue effects included shoulder stiffness and arm oedema, with the contralateral side used for comparison. In START-A and B, patient-reported assessments of late normal tissue effects in the arm and shoulder were collected using items from the EORTC QLQ-BR23 [12] and protocol-specific questions relating to post-radiotherapy changes. Patients completed the EORTC QLQ-BR23 at baseline, 6, 12, 24 and 60 months after randomisation; the protocol items relating to post-radiotherapy changes were collected at 6, 12, 24 and 60 months. All physician and patient-reported assessments of late normal tissue effects were scored on a 4-point scale (none, a little, quite a bit, very much). Brachial plexopathy was reported if damage to the brachial plexus was suspected and the patient had symptoms of pain, paresthesia, numbness, or other sensory symptoms (graded on a 4-point scale). Suspected cases of brachial plexopathy were subject to confirmation by neurophysiological assessment and MRI.

The START trials are registered as an International Standard Randomised Controlled Trial, number ISRCTN59368779.

## Statistical methods

All normal tissue effect assessment scores (patients and physicians) were dichotomised as “none/a little” versus “quite a bit/very much” (interpreted as none/mild versus moderate/marked effects).

Survival analysis methods were used to investigate time to first moderate or marked effect, using the date of completion of the PROMS questionnaire or date of annual follow-up visit to calculate length of follow-up from randomisation. The Kaplan–Meier estimates of cumulative incidence rates of moderate/marked effects for each fractionation schedule were obtained (with 95% confidence intervals, CI). Hazard ratios (HR, with 95%CI) for each test schedule compared with the control group were obtained from Cox proportional hazards regression analysis using all available follow-up data. For symptoms included in the baseline PROMS questionnaire, the Cox’s proportional hazards regression model included a term for the baseline score. The proportional hazards assumption was checked using the Schoenfield residuals.

As events reported at earlier follow-up may potentially be post-surgical rather than radiotherapy effects, cross-sectional analyses were also done, focussing on the point prevalence of moderate/marked effects at 5 years (with exact 95% confidence interval, CI), and each test schedule compared with the control group using Chi-squared or Fisher’s exact test where appropriate. Ten-year cross-sectional analyses were also done for the physician assessments.

Corresponding survival analyses for arm and shoulder effects in patients who received only breast/chest wall radiotherapy in the START trials were included in forest plots for comparison with the lymphatic radiotherapy group.

Each trial was analysed separately, but no subgroup analyses were done due to the small number of patients and events in some groups. There was no formal adjustment for multiple testing.

Analysis was on an intention-to-treat basis, as compliance with the randomised treatment was high in the trials.

## Results

Overall 864/5861 (14.7%) patients across all three trials were treated with lymphatic radiotherapy and included in this analysis. This includes 385/1410 (27.3%) in the START-pilot trial, 318/2236 (14.2%) in START-A and 161/2215 (7.3%) in START-B. Of these, physician assessments of normal tissue effects were available for 298/385 (77.4%) in the pilot trial, 304/318 (95.6%) in START-A and 154/161 (95.6%) in START-B. Patient-reported assessments of normal tissue effects were available for 250/262 (95.4%) patients in START-A who received lymphatic radiotherapy and similarly for 98/103 (95.1%) in START-B. Median follow-up for the patients included in the analysis was 10 years.

There were differences between patient characteristics of those who received lymphatic radiotherapy in the three trials (Table 1). The majority of patients in the START-pilot trial received RT to both the axilla and SCF (75.0%), compared with 9.2% in START-A and 13.0% in START-B. Lymphatic RT to the axilla only was received by 0.3% in each of the START-pilot and START-A trials and 29.8% in START-B, and by 24.7%, 90.5% and 57.2% to SCF only in the START-pilot, A and B trials respectively. More patients received axillary surgery in START-A (97.2%) and START-B (95.1%) than in the START-pilot trial (39.6%). Patients were only eligible for the START-pilot trial following breast conserving surgery; START-A and B included mastectomy, with more in START-A (46.2%) compared with START-B (34.2%). Tumour grade was not available for all patients (particularly in the START-pilot trial), but tended to be lower for patients in START-B (112 grade 1 or 2 out of 158, 70.9%) compared with 51.5% (34/66) in the START-pilot and 58.4% (184/315) in START-A out of those for whom grade was known. Adjuvant therapies also varied, with 37.7% in the START-pilot trial receiving tamoxifen only, compared with 18.6% in START-A and 41.6% in START-B, and more patients receiving chemotherapy in trial A compared with trial B. Fewer patients in START-B received a breast radiotherapy boost (51.9%) compared with 73.8% in the pilot and 83% in START-A.

**Table 1:** Baseline and treatment characteristics of patients who received lymphatic radiotherapy in the START-pilot, START-A and START-B trials.

	START-pilot Total *n* = 385 (%)	START-A Total *n* = 318 (%)	START-B Total *n* = 161 (%)
*Age (years)*			
Median (IQR) [range]	52.4 (45.5–60.5) [25.4–78.5]	56.2 (48.7–65.4) [25.7–81.9]	56.6 (50.7–65.2) [24.7–86.8]

*Primary surgery*			
Breast conserving surgery	385 (100.0)	171 (53.8)	106 (65.8)
Mastectomy	0	147 (46.2)	55 (34.2)

*Histological type*			
Invasive ductal	297 (77.1)	256 (80.5)	126 (78.3)
Invasive lobular	27 (7.0)	42 (13.2)	26 (16.2)
Mixed ductal/lobular	14 (3.7)	10 (3.1)	2 (1.2)
Other	47 (12.2)	8 (2.5)	6 (3.7)
Not known	0	2 (0.7)	1 (0.6)

*Pathological node status*			
Positive	129 (33.5)	274 (86.2)	144 (89.4)
Negative	21 (5.5)	34 (10.7)	9 (5.6)
Not known (no axillary surgery)	233 (60.5)	9 (2.8)	8 (5.0)
Not known (missing data)	2 (0.5)	1 (0.3)	0

*If positive, number of involved nodes*			
Median (IQR) [range]	2 (1–5) [1–19]	3 (1–6) [1–25]	3 (1–6) [1–23]

*Tumour size (cm)*			
<1	7 (1.8)	12 (3.8)	3 (1.9)
1-	109 (28.3)	105 (33.0)	49 (30.4)
2-	106 (27.5)	111 (34.9)	61 (37.9)
3-	85 (22.1)	86 (27.0)	47 (29.2)
Not known	78 (20.3)	4 (1.3)	1 (0.6)

*Tumour grade*			
1	8 (2.1)	28 (8.8)	31 (19.3)
2	26 (6.7)	156 (49.1)	81 (50.3)
3	32 (8.3)	131 (41.2)	46 (28.6)
Not known (missing data)	319 (82.9)	0	1 (0.6)
Not known (not applicable*)	0	3 (0.9)	2 (1.2)

*Adjuvant therapy*			
None	0	5 (1.6)	4 (2.5)
Tamoxifen only	145 (37.7)	59 (18.6)	67 (41.6)
Chemotherapy only	19 (4.9)	56 (17.6)	16 (9.9)
Tamoxifen + chemotherapy	44 (11.4)	184 (57.9)	74 (46.0)
Other endocrine therapy**/Not known	177 (46.0)	14 (4.3)	0

*Lymphatic treatment*			
Surgery + Axilla only	1 (0.3)	0 (0.0)	48 (29.8)
Surgery + SCF only	93 (24.2)	285 (89.6)	90 (56.0)
Surgery + Axilla + SCF	58 (15.1)	24 (7.6)	15 (9.3)
No surgery + Axilla only	0 (0.0)	1 (0.3)	0 (0.0)
No surgery + SCF only	2 (0.5)	3 (0.9)	2 (1.2)
No surgery + Axilla + SCF	231 (59.9)	5 (1.6)	6 (3.7)

*Boost (BCS patients only)*			
Yes	284 (73.8)	142 (83.0)	55 (51.9)
No	101 (26.2)	26 (15.2)	51 (48.1)
Not known	0	3 (1.8)	0

IQR, interquartile range; SCF, supraclavicular fossa; BCS, breast conserving surgery.

* Lobular and other histological types.

** Other endocrine therapies include combinations of tamoxifen/anastrozole/exemestane/goserelin, mostly within randomised trials.

### Survival analyses

Cumulative incidence rates of patient-assessed moderate/marked effects in the arm or shoulder up to 5 years were similar between the test and control schedules in START-A and START-B (Table 2). The number of cumulative events per schedule was small for many of the outcomes, reflected in the wide CIs. In the 50 Gy control groups, rates were highest for arm/shoulder pain (32.3%, 95%CI 23.3–43.7 by 5 years in START-A) and lowest for arm/hand swelling (9.5%, 95%CI 3.7–23.3 by 5 years in START-B). None of the hazard ratios indicated a statistically significant difference in patient-assessed arm or shoulder effects between the hypofractionated schedules and the 50 Gy control groups for either START-A or START-B.

**Table 2:** Patient-assessed moderate/marked normal tissue effects in the arm or shoulder following lymphatic radiotherapy in START-A and START-B.

^1^Results adjusted for baseline; *P*-values represent comparison of each test schedule with 50 Gy; ^2^Wald test; ^3^Fisher’s exact test.

For physician-assessed moderate/marked arm oedema or shoulder stiffness, cumulative incidence rates up to 5 and 10 years were generally similar between the test and control schedules in the START-pilot, START-A and START-B trials, with few events reported in most categories (Table 3). There was a statistically significant increased rate of physician-assessed shoulder stiffness in the 42.9 Gy schedule compared with 50 Gy in START-pilot (HR 3.07, 95%CI 1.62–5.83, *p* = 0.001) but no such effect for the hypofractionated schedules in START-A and START-B. There were no statistically significant differences in physician-assessed moderate/marked arm oedema between the schedules for any of the trials. There was generally little increase in effects over time, with 5- and 10-year cumulative incidence rates for physician-assessed arm oedema and shoulder stiffness broadly similar.

**Table 3:** Physician-assessed moderate/marked normal tissue effects in the arm or shoulder following lymphatic radiotherapy in START-pilot, START-A and START-B.

*P*-values represent comparison of each test schedule with 50 Gy; ^1^Wald test; ^2^Fisher’s exact test.

**Fig. 1:**
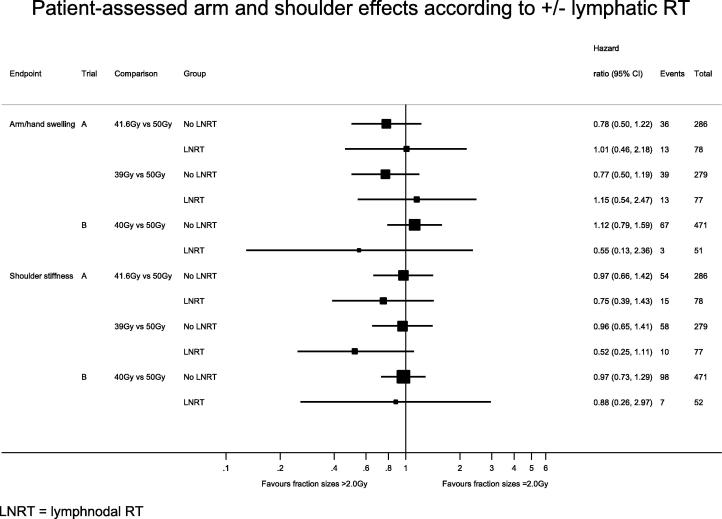
Patient-assessed arm and shoulder effects according to ± lymphatic RT. RT, radiotherapy; LNRT, lymph nodal radiotherapy.

**Fig. 2:**
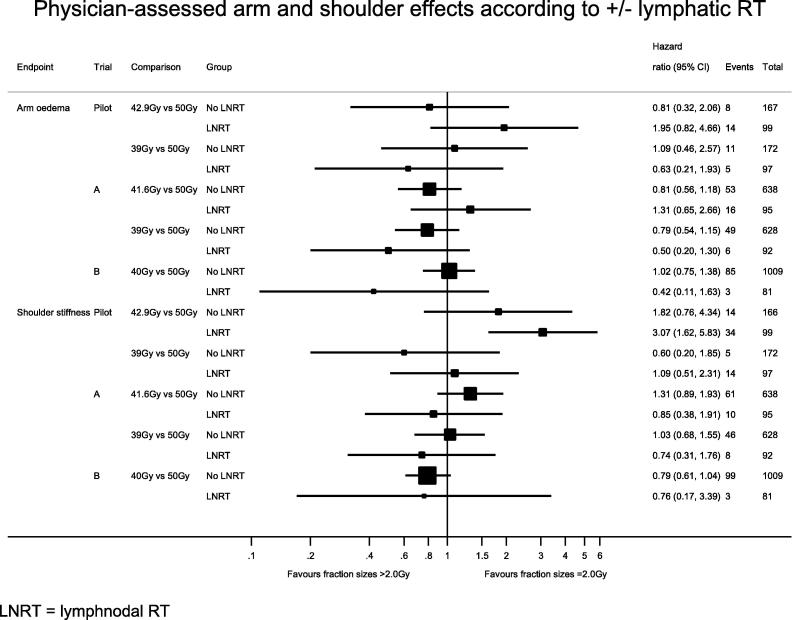
Physician-assessed arm and shoulder effects according to ± lymphatic RT. RT, radiotherapy; LNRT, lymph nodal radiotherapy.

Using the patients in the START trials who only received breast/chest wall RT as a comparison group, there was no evidence of a statistical difference in hazard ratios for the hypofractionated schedules compared with 50 Gy according to whether or not lymphatic RT was given (results for arm oedema and shoulder stiffness are shown in Fig. 1, Fig. 2 for patient and physician assessments respectively).

### Cross-sectional analyses

At 5 years following radiotherapy, the point prevalences of moderate/marked effects in the arm or shoulder reported by patients and physicians were low overall, with very few events and no statistically significant differences between the hypofractionated and control schedules for any of the trials (Table 2, Table 3). This remained so for the physician assessments at 10 years (Table 3). The 5- and 10-year point prevalences were much lower than the estimates of cumulative incidence up to 5 and 10 years for all of the effects.

## Discussion

Our investigation of late normal tissue effects in the arm and shoulder for women treated with locoregional RT within the START trials suggests that appropriately-dosed hypofractionated lymphatic irradiation is comparable to the traditional normofractionated (2.0 Gy) schedule in terms of safety. Adverse event rates were low overall, and point prevalences at 5 and 10 years were generally considerably lower than cumulative incidence rates, partly due to reversal of post-surgical effects reported early in follow-up. Although we have not carried out formal tests of interaction due to small sample sizes in subgroups, comparing results of relative treatment effects between patients with and without lymphatic RT in the START trials showed no evidence of differential effects of the hypofractionated schedules compared with the control schedule of 50 Gy in 25 fractions, supporting our conclusions. This is an important point, since it suggests that arm oedema and shoulder stiffness are no more sensitive to fraction size than breast/chest wall toxicity endpoints. Thus, the higher hazard ratios for arm oedema and shoulder stiffness reported in the START-pilot trial after 13 fractions of 3.3 Gy compared with 50 Gy in 25 fractions are not surprising given that this test dose level is equivalent to prescribing 54 Gy in 2.0 Gy fractions, assuming an alpha/beta ratio of 3 Gy.

A comparison of these results with published data is not straightforward as various studies define and measure arm and shoulder normal tissue effects in distinct ways and at different time points. Evaluation method and time interval from treatment have an impact on arm oedema scores, as reported in a systematic review of the evidence related to lymphedema in breast cancer patients [13]. In the review, clinical diagnosis by physicians resulted in 12.6% incidence of lymphoedema compared with self-reported swelling in 20.4%. In the EORTC 10981-22023 AMAROS trial testing radiotherapy versus surgery of the axilla after a positive sentinel node biopsy, lymphoedema was reported less often when defined as an increase in arm circumference of ≥10% (6% rate at 5 years) compared with clinician evaluation based on presence of ‘any’ signs of arm oedema (11% rate at 5 years) [14]. The systematic review and the AMAROS trial both suggested that incidence of arm oedema tended to increase in the first 1 or 2 years following diagnosis or surgery, and then to decrease [13], [14]. It is reassuring that the START data show no relative differences between the schedules, however it is likely that absolute rates of normal tissue effects are now lower using modern target volume-based RT compared with the field-based RT used in the era of the START trials.

In the START trials radiotherapy-related adverse effects in the arm and shoulder were assessed using both patient and physician assessments, each based on a 4-point scale. Although patients report higher absolute event rates than physicians, as previously described for breast adverse effects [15], the overall conclusions from the comparison of schedules are consistent, strengthening the conclusions from this retrospective analysis that there is no evidence of a detrimental effect of the hypofractionated schedules on arm and shoulder symptoms. The importance of the PROMS is re-enforced by data suggesting a correlation between functional symptoms, including shoulder stiffness and arm/shoulder pain, and quality of life indices in women treated with conservative surgery and radiotherapy for breast cancer [16]. In our study, the 5- and 10-year prevalence data from physician assessments show that lymphoedema rates are relatively stable at these time points, suggesting the safety of hypofractionated lymphatic radiotherapy in the long-term.

Due to differences between the START-pilot, A and B trials, the patient sample included in this retrospective analysis is heterogeneous in terms of proposed risk factors for arm and shoulder toxicity, i.e. axillary treatment, extent of surgery, adjuvant systemic therapy and radiotherapy technique. This variation does not impact on the comparative analysis between normofractionated and hypofractionated schedules however, as these variables are well-balanced amongst randomised groups in each trial [2], [3], [4]. The main limitation of this retrospective analysis is the relatively small sample size of the lymphatic radiotherapy subgroups in each trial and the low rates of reported late normal tissue events in the arm and shoulder, which limit the statistical power of the analyses. Additionally, due to the low locoregional relapse rates in the START trials overall, the number of events prohibited reliable statistical analysis of the efficacy of hypofractionated lymphatic radiotherapy in terms of tumour control in this subgroup analysis.

Radiobiological estimates of equivalent total dose in 2.0 Gy fractions (EQD_2Gy_) for the tested hypofractionated schedules with regard to brachial plexus toxicity [17] raise no specific concerns with regard to the brachial plexus. Based on the START trials, the EQD_2Gy_ of 40 Gy in 15 fractions is 46 Gy and 48 Gy, assuming alpha/beta values of 3 Gy and 1.5 Gy, respectively. One patient in the START trials developed mild symptoms and signs of brachial plexopathy two years following treatment to the breast and supraclavicular fossa on the 41.6 Gy schedule in START-A. She had a family history of polydactyly (accessory thumb) on the affected side, raising the possibility of a yet-to-be-identified genetic predisposition. Lymphatic radiotherapy is now volume-based and doses are prescribed to more relevant reference points [18]. In the START-pilot trial, lymphatic radiotherapy comprising an anterior field to the supraclavicular fossa was prescribed as an applied dose. If the axilla was included, an equally weighted posterior axillary field was treated with every fraction to ensure that 100% of the prescribed dose was delivered to the axillary midline [2]. In the START-A and B trials, a posterior field, weighted according to axillary separation was adopted if the mid-axilla dose fell below 80% of the applied dose. A reassuring point is that fractionation sensitivity will not change, even though volume coverage, dose intensity and homogeneity may do so. If radiation oncologists are confident in prescribing 50 Gy in 25 fractions to contemporary lymphatic volumes and reference points, the START analysis presented here suggests they can be equally confident in prescribing appropriately-dosed hypofractionation.

In order to address residual concerns regarding extensive adoption of moderate hypofractionation in women undergoing radiotherapy for breast cancer worldwide, five randomised trials have been launched in the last two years in Denmark, US, Egypt and France. The largest trial (target *N* = 2000) is sponsored by the Danish Breast Cancer Cooperative Group (ClinicalTrials.gov identifier: NCT02384733), testing 40 Gy in 15 fractions versus 50 Gy in 25 fractions in terms of late normal tissue effects and tumour control in patients treated with mastectomy or breast conserving surgery for pT1–3, pN0–3, M0 invasive breast cancer with indications for radiotherapy to regional lymph nodes. The other four randomised trials (ClinicalTrials.gov identifiers: NCT02690636, NCT02700386, NCT02958774, NCT03127995) have a similar design, with the test group receiving 15 or 16 fractions of 2.7 Gy and the control group receiving the normofractionated 25 fraction schedule. Internal mammary node irradiation, which was not permitted in the START trials, is also being investigated in two of these trials.

Shorter course hypofractionation for lymphatic radiotherapy is being investigated in a sub-study of the UK FAST-Forward randomised trial (ISRCTN19906132), which compares two 5-day schedules (27 Gy in 5 fractions of 5.4 Gy and 26 Gy in 5 fractions of 5.2 Gy) with 40 Gy in 15 fractions over 3 weeks (control).

Within the next few years, these randomised trials will produce long-term data on locoregional tumour control and toxicity in over 3700 women treated with hypofractionated lymphatic radiotherapy. In the meantime, whilst bearing in mind the statistical limitations, the long-term results from this retrospective subgroup analysis of the START trials suggest that appropriately-dosed hypofractionated lymphatic radiotherapy is safe, a conclusion consistent with the findings for >2.0 Gy schedules delivered to the breast/chest wall.

## Role of funding source

The funders had no role in the study design or conduct, analysis or interpretation of data, manuscript writing or the decision to submit for publication.

## Conflict of interest statement

The authors declare no conflicts of interest.
